# Effect of Short-Term Deep-Pressure Portable Seat on Behavioral and Biological Stress in Children with Autism Spectrum Disorders: A Pilot Study

**DOI:** 10.3390/bioengineering9020048

**Published:** 2022-01-20

**Authors:** Ilham Yustar Afif, Muhammad Farkhan, Ojo Kurdi, Mohamad Izzur Maula, Muhammad Imam Ammarullah, Budi Setiyana, J. Jamari, Tri Indah Winarni

**Affiliations:** 1 Undip Biomechanics Engineering & Research Centre (UBM-ERC), Diponegoro University, Semarang 50275, Indonesia; iyustar.afif@gmail.com (I.Y.A.); izzurmaula@gmail.com (M.I.M.); imamammarullah@gmail.com (M.I.A.); j.jamari@gmail.com (J.J.); 2 Department of Mechanical Engineering, Faculty of Engineering, Diponegoro University, Semarang 50275, Indonesia; mfarkhan43@gmail.com (M.F.); ojokurdi@ft.undip.ac.id (O.K.); bsetiyana@yahoo.com (B.S.); 3 Department of Anatomy, Faculty of Medicine, Diponegoro University, Semarang 50275, Indonesia; 4 Center for Biomedical Research (CEBIOR), Faculty of Medicine, Diponegoro University, Semarang 50275, Indonesia

**Keywords:** anxiety, autism spectrum disorders, behavioral problems, deep pressure, hug machine

## Abstract

Children with autism spectrum disorder (ASD) have challenging behaviors, which are associated with difficulties in parenting. Deep pressure is a therapeutic modality in occupational therapy, and it was reported to produce a calming effect. This study aimed to determine whether the short-term use of an autism hug machine portable seat (AHMPS) improves behavioral and neurobiological stress in children with ASD, and to determine whether AHMPS with an inflatable wrap or manual pull is more effective. This study enrolled children with ASD who were administered with the inflatable wrap (group I) and manual pull (group II) for 20 min twice a week for 3 weeks. Conners’ Parent Rating Scale-48 (CPRS-48) was used to rate behavioral improvements, and galvanic skin response (GSR) was used to measure sympathetic stress response. A total of 20 children with ASD (14 boys and 6 girls; aged 7–13 years) were included. CPRS-48 presented conduct problems: behavior was significantly decreased in the inflatable group (*p* = 0.007) compared to the manual pull group. The GSR captured a significant reduction in sympathetic response (*p* = 0.01) only in group I. Neurobiological stress was reduced in children who were wearing the AHMPS inflatable wrap; therefore, AHMPS inflatable wrap is an effective method to reduce emotional arousal.

## 1. Introduction

Autism spectrum disorder (ASD) is a developmental disorder that affects communication and behavior. According to the *Diagnostic and Statistical Manual of Mental Disorders* (*DSM-V*), individuals with ASD have three characteristics, namely, difficulty with communication and interaction with other people, restricted interests, and repetitive patterns of behaviors, and sensory integration disorders (SIDs) that affect an individual’s ability to function properly [1]. The recent ASD prevalence reported by a review of Chiarotti et al., (2020) in 11 states of the USA has significantly increased; in 2014, autism was estimated to occur in 16.8/1000 children aged 8 years and increased to approximately 18.5/1000 children in 2016 [2]. The Central Bureau Statistics of Indonesia predicted that 1.14% of the population (2.4 million individuals) had autism in 2018 (unpublished data). Genetic, epigenetic, environmental, and immunological factors are linked to the pathophysiology of autism and affect the neuroanatomical and neurochemical development of the central nervous system [3,4,5]. Single-gene disorder is associated with the etiology of autism, which accounts for 20–30% of cases [3]. Duffney et al. concluded that approximately 215 genes with an epigenetic-modulating function are involved in 19.5% of autism cases [6]. The presence of brain-reactive maternal autoantibodies was found in another 20% of autism cases. Recently, dysregulation of the maternal immune system in gestation has been found to play an important role in the changes in neurodevelopment, leading to the development of neurobehavioral disorders, called maternal autoantibody-related autism [7].

Although a previous study confirmed that neurochemical dysregulation underlines autism symptoms, the pathophysiology is still unclear. Jones et al. reported the association between maternal autoimmunity and the acute onset of neuropsychiatric disorders or offspring global regression, including ASD, due to the alteration of fetal microglia (brain’s resident cells that regulate brain development, maintenance of neuronal networks, and injury repair) or the altered transcription of neuro-developmental vulnerability/immune genes in utero by multiple mechanisms, including epigenetic, the direct effect of maternal immune molecules, and priming microglia affecting synaptic development, and the potential further activation ex utero by stress and infection [8,9]. Another study concluded that an excitatory (glutamate-mediated) and inhibitory (gamma-aminobutyric acid/GABA-mediated) neuron imbalance in the brain is responsible for the autism phenotype [10]. A reduction in the levels of GABA, the main inhibitory neuron that has a crucial role in sensory integration (SI), has been reported consistently in children with autism compared with children with typical development [11]. Therefore, most children with ASD have an impaired sensory input, such as sound, touch, body movement/position, sight, taste, and smell; this results in a behavioral response to the sensory input experienced, which is called sensory processing (SP) difficulty. SP disorders are common in autism, with prevalence estimates of 90% [12], and they are associated with a wide variety of difficulties across many domains, that is, externalizing and internalizing behaviors; emotional and attention regulation; executive function; and daily-living functional activities, including social activities [13,14]. SP difficulties in children can have an impact on their participation in daily life activities and enjoyable tasks because of their atypical reactions to sensory stimuli and their impaired self-esteem [15]. Therefore, traveling with children with SP disorders can be very challenging for parents and a stressful event for the child. It can lead to behavioral problems, such as verbal tantrums (crying, arguing, screaming, and shouting) and physical tantrums (defiant, kicking, banging toys, and throwing), as well as self-injurious behavior affecting not only their own safety but also that of others, including the driver. Traveling using public transportation can be even worse because of many people being inside the same vehicle (social engagement-threatening situation), unpredictability, and crowds, and the environment can be very noisy [16].

SP disorders are frequently reported, and given the widespread impact on daily life skills and occupational performance, various sensory-based intervention (SBI) options have been recommended to address the difficulties. Parents reported that occupational therapy using the SBI approach, including a weighted vest and a pressure vest, is important and helpful when addressing challenging behaviors [17]. Deep pressure as a therapeutic method in occupational therapy was first reported to produce a calming effect through the modulation of central nervous system (CNS) sensory information processing [18]. In the 1990s, Grandin developed a squeeze machine, which was originally created as a stress-relieving device to calm him down. The principle of this device is to give “a hug” stimulation, whose deep-pressure effect calms the user. This tool was developed based on his own experience as a patient with ASD, who frequently experienced arousal, including anxiety and hyperactivity [19]. Autistic individuals commonly display hypersensitivity to touch. The perception of touch is sensed in the skin by four subtypes of low-threshold mechanoreceptors (LTMRs) and small-diameter, lightly/unmyelinated neurons (C fibers), which are responsive to pain and positively correlated with touch pleasantness; therefore, these are implicated in social perception and the recognition process in individuals with ASD [20]. The deep pressure signal stimulates the brain through the dorsal column system of the spinal cord, followed by ascending to the brain via ascending pathways, including the spinoreticular pathway, which contributes to autonomic activity [21]. In addition, the deep-pressure organized sensory input stimulates the release of serotonin and dopamine, which influence deep brain structures, decreasing arousal [22,23].

The hug machine, with an adjustable strength pressure or intensity, was developed from the hugging theory; this theory can be used to develop a portable device that helps mobilization in children with autism [23]. Moreover, it will bring beneficial effects to the development of the medical device in emerging countries, especially in Indonesia, that have problems with costs related to the procurement of medical devices for the purpose of meeting market needs [24,25,26,27,28,29,30,31]. Emotional and physiological arousal is regulated by the autonomic nervous system, which is principally sustained by parasympathetic- and sympathetic-balanced activities. Skin conductance has been widely used to measure the activity of the autonomic nervous system [32], and it is reported as a very sensitive and valid biomarker of non-invasive emotional stress/arousal. A fluctuation in the activity of the sympathetic nervous system stimulates the sweat glands to secrete sweat intensively [33]. This study aimed to determine the effect of the short-term use of an autism hug machine portable seat (AHMPS) to improve behavioral and neurobiological stress in children with ASD and to measure the effectiveness of AHMPS with an inflatable wrap compared to the manual pull model.

## 2. Materials and Methods

### 2.1. Study Design and Population

This single-blind experimental study with pretest and posttest design was conducted at Public Special School, Central Java, Semarang, Indonesia. Participants were children diagnosed with ASD by a physician or psychologist using *DSM-IV* criteria. Children with autism were randomized into two groups, i.e., group I, which was administered with an autism hug machine portable seat (AHMPS) with an inflatable wrap, and group II, which was administered with an AHMPS with a manual pull. Therapy sessions were delivered in the morning to avoid an exhausting experience with peers, and they were carried out by researchers and trained education staff in a regular classroom with minimal distraction.

### 2.2. Instruments

#### 2.2.1. Autism Hug Machine Portable Seat (AHMPS)

**Figure 1:**
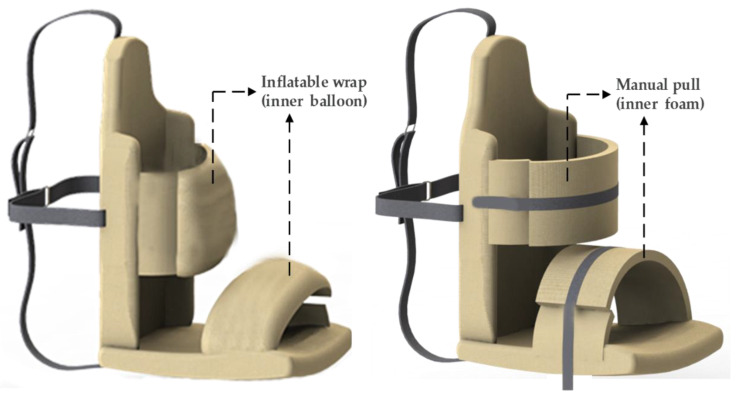
Model of AHMPS inflatable wrap (**left**) and manual pull (**right**).

Two models of an AHMPS designed from a previous study were used: an AHMPS with an inflatable wrap and an AHMPS with a manual strap as shown in Figure 1 [34]. The pressure load was determined based on a qualitative comfort test in 15 adult healthy and asymptomatic individuals, conducted in a previous study [35]. Healthy asymptomatic individuals were recruited to ensure communication and an objective judgment [36]. The AHMPS with a manual pull is a hug machine with hugging straps in the form of soft foam coated with leather fabric. The deep-pressure effects are established when the straps are pulled manually to tighten it and press the body parts, while the inflatable wrap is a hug machine with a balloon-type hugging strap coated with leather fabric. In group I, the pneumatic pressure technique, a modified form of the technique of Grandin et al. [19], was employed, where the inflating wrap pressure was measured using the MPX5500DP^®^ (Austin, TX, USA) air pressure sensor. In group II, the pressure was measured using load sensors installed along with the straps, which were in contact with the user’s body.

#### 2.2.2. Conners’ Parent Rating Scale-48 (CPRS-48)

CPRS-48 has been used to measure behavioral abnormalities [37]. The scale was shortened substantially and reworded in order to simplify administration and interpretation. The remaining items of the parent questionnaires were only items that loaded on each factor for both parents, forming identical factors by both mother and father ratings. Thus, a psychometric measurement tool composed of a 22-item pool using a 4-point Likert scale was used, and scores ranged from 0, indicating “not at all true”, to 3, indicating “very much true”. This validated short version provided quantitative and qualitative representations of children’s emotions and behavior based on five subscales, i.e., conduct problems (items 2, 8, 14, 19, 20, 27, 35, and 39), learning problems (items 10, 25, 31, and 37), psychosomatic problems (items 32, 41, 43, and 44), impulsive–hyperactive behaviors (items 4, 5, 11, and 13), and anxiety (items 12 and 16) [37]. Parents were asked to rate their children’s behavior using CPRS-48 before the therapy session began and immediately after the last therapy session was finished.

#### 2.2.3. Galvanic Skin Response (GSR)

Neurobiological stress response was measured using GSR to capture skin conductance variability. Changes in sympathetic innervation activation, particularly the activities of the sweat glands in the skin, were measured as a tonic and phasic fluctuation in electrodermal activity using a skin conductance device [33]. GSR electrodes were placed on the index and middle fingers of the right hand by Velcro strap, and after 15 s, the skin conductance was displayed on screen. The skin conductance of children with ASD in both groups was measured three times, i.e., before, during, and after the intervention.

### 2.3. Procedures

A portable AHMPS was installed on the chair by looping the AHMPS straps onto the seat using the Velcro strap on the back of the AHMPS. The straps were then tied from the right to the left side and from the top to the bottom side of the chair. To familiarize the child with the devices, i.e., AHMPS and GSR electrodes, presence of the researcher, and the experimental procedure, a pretrial session was carried out; that is, the child was placed on the portable seat for 20 min before being assigned to the experimental condition. This session also aimed to ensure that all children could tolerate the pressure load prior to the experimental session.

The therapy session was carried out during the morning class session (at 9–10 am) in a regular classroom and carried out by applying AHMPS straps to the chest and thighs. In group I, the inflatable wraps were inflated by pressing the control button on the automatic pressure control system. The air pressure was set at 4.48 kPa (0.65 psi) on the chest and 3.10 kPa (0.45 psi) on the thighs. The sensor system was turned off when the set pressure was reached. In group II, load sensors were fixed on the child’s chest and thighs before the straps were placed, and the straps were then pulled manually by the researcher or caregiver until the pressure sensor reached 5.58 kPa (0.81 psi) on the chest and 5.52 kPa (0.80 psi) on the thighs. The pressure load was determined based on a previous study [35]. Therapy session was delivered for 20 min twice a week for 3 consecutive weeks, and each participant had six sessions [38].

### 2.4. Data Analysis

Child behaviors and skin conductance variability data were normally distributed with a significance level of 0.05. A *t*-test was carried out to determine the behavioral difference between pretest and posttest in both the inflatable group and manual group. Furthermore, an independent sample t-test was carried out to analyze the behavioral improvement in each CPRS-48 subscale, i.e., conduct problems, learning problems, psychosomatic problems, impulsive–hyperactive behaviors, and anxiety, between the inflatable group and manual group. The effect size (r) was low if the value of r was approximately 0.1, medium if r was approximately 0.3, and large if r was more than 0.5. A repeated measurement test was performed to measure the effect of the intervention on decreasing neurobiological stress response using GSR in both groups. The effect size of the intervention was measured using partial eta squared (η_p_^2^), where η_p_^2^ = 0.1 indicated a low effect, η_p_^2^ = 0.3 indicated a medium effect, and η_p_^2^ = 0.5 indicated a large effect. A *p*-value < 0.05 indicated statistical significance. IBM SPSS software program was used to conduct the data analysis [39].

## 3. Results

This experimental study enrolled 20 children with ASD, including 14 boys and 6 girls aged 7–13 years; the mean age was 9.6 ± 2.55 years in group I, and the mean age of group II was 10.1 ± 2.56 years. All participants were randomly assigned into group I and group II, and each group was composed of 10 children. All participants were Javanese, and participants in both groups were matched for age (*p* = 0.66) and gender (*p* = 0.36).

**Figure 2:**
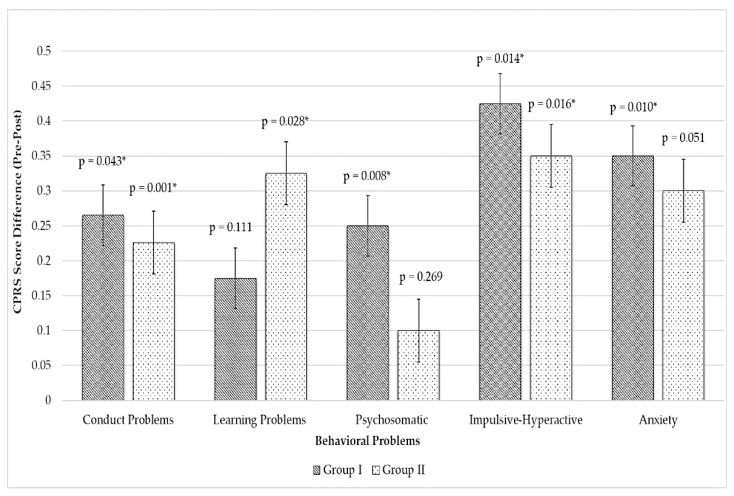
Parents’ rating of behavioral improvement (pretest–posttest) among children in the inflatable group (group 1) and manual group (group II). Data with * indicate a statistically significance.

Child behavior data are shown in Figure 2. Intragroup data revealed that group I demonstrated significant improvement in conduct problems, psychosomatic problems, impulsive–hyperactive behaviors, and anxiety, while group II exhibited significant improvement in conduct problems, learning problems, and impulsive–hyperactive behaviors.

Table 1 shows the pretest and posttest scores (mean ± SD) on the subscales of CPRS-48: conduct problems, psychosomatic problems, impulsive–hyperactivity behavior, and anxiety in both groups. An independent t-test was carried out to determine the difference in behavioral improvement in both groups. Only conduct problems were significantly decreased in group I compared with group II (*p* = 0.007, η_p_^2^ = 0.48).

**Figure 3:**
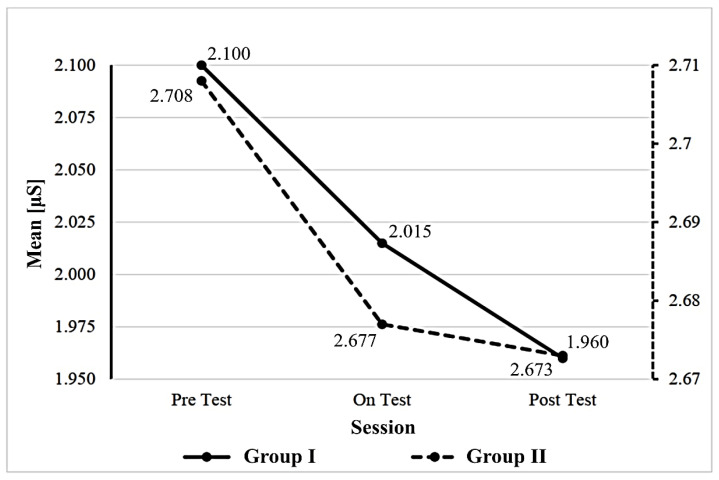
Improvement in stress response using galvanic skin response tool in group I (continuous line) and group II (dashed line) in a pre–on–post experiment.

The emotional arousal or stress variability of each child in both groups was captured using GSR three times, i.e., pretest, during, and posttest, and the results are presented in Figure 3. A repeated measurement test was carried out to measure the effectiveness of the automatic inflating wrap, and it revealed that an AHMPS with an inflatable wrap significantly reduced emotional stress (*p* < 0.011) in children with autism. Psychophysiological stress was decreased by deep pressure using an automatic inflating wrap (*F* (1.6, 14.16) = 5.90, *p* = 0.011) with a medium effect (η_p_^2^ = 0.396). In group II, the reduction in emotional arousal was not caused by wearing a portable seat with a manual pull (*F* (1.9, 16.7) = 0.946, *p* = 0.407) with a small effect (η_p_^2^ = 0.09).

## 4. Discussion

The parents of children in both groups reported behavioral improvement, except for learning problems in group I and psychosomatic behaviors in group II. However, only conduct problems improved significantly (*p* = 0.046) in group I compared with group II. The conduct problem subscale was composed of eight items of externalizing and internalizing behaviors, i.e., sassy to grown-ups, angry all the time, destructive mood, denies mistakes/blames others, quarrelsome, bully others, fights constantly, and unhappy. The parents of children with ASD often face emotional and behavioral disorders with their children, especially when the child is overwhelmed with sensory stimulation [40]. In the late 1980s, a study reported the effect of deep-pressure touch as an occupational therapy to provoke a calming effect in a child and an adult with anxiety with hyperactivity manifestation through parasympathetic nerve escalation [41,42]. Deep pressure has been proven to decrease sympathetic arousal, increase parasympathetic response, and improve behavioral performance [41].

In our study, the AHMPS with the inflating wrap was found to significantly reduce emotional arousal in children with autism. The reduction in psychophysiological stress was due to the medium effect of the deep pressure generated by the automatic inflating wrap. In group II, the improvement in emotional arousal using GSR was not caused by the pressure from the manual pull. A higher pressure was administered in group II than in group I. Deep pressure, a sensory stimulation therapy that results in comfort, is widely used to overcome SID. Group II received higher pressure than group I, which may stimulate discomfort. Hein et al. found that discomfort stimulates the sympathetic nervous system, which results in an increased heart rate, peripheral vasoconstriction, and an increased skin conductance response [43,44]. The deep-pressure effect is also influenced by a large contact area: the larger the contact area, the greater the benefits [19,35]. The inflatable wrap provides pressure in all directions so that it can reach areas of the body that are not in contact with the manual pull. A similar experiment was carried out by Minoura et al., who replaced the foam pads with bead-filled cushions, which can cover a large surface area and provide a better relaxing effect [45].

This study was only conducted in children with ASD who were enrolled in a special school, and some routine interventions were provided by the school. The parents’ cognitive and behavioral factors, such as perception, acquiescence, expectancy, conditioning, suggestion, and motivation, were not measured in this study, which may have influenced the effect of the CPRS-48 measurement [46]. A comparison between the teachers’ and parents’ behavioral rating scales would be very interesting, as it would show the effect of the hug machines, which may be a useful therapy option to be used at school; however, we did not ask the teachers to rate the children’s behaviors. The duration of the AHMPS effect was not calculated in this study, and so the duration of the decreased neurobiological stress and improved behavior cannot be reported.

Future studies should be carried out to investigate the duration and intensity of the intervention. It is important to determine the most appropriate duration and frequency for the intervention to guide intervention recommendations. It would be interesting to include the teachers’ rating scale in future studies.

## 5. Conclusions

The AHMPS with an inflatable wrap improves emotional arousal behavior in children with ASD. Parents also reported a reduction in behavior problems in children using the inflating wrap and manual pull. Additional studies are needed to observe more biological outcome measures and to confirm the effectiveness of the AHMPS.

## Figures and Tables

**Table 1:** The five subscales of Conner’s Parent Rating Scale-48 score of groups I and II.

Subscales	Group	N	Pretest (Mean ± SD)	Posttest (Mean ± SD)	Mean Difference	*p*
Conduct problems	I	10	1.441 ± 0.442	1.176 ± 0.158	−0.482	0.007 *
	II	10	1.866 ± 0.498	1.640 ± 0.435		
Learning problems	I	10	2.250 ± 0.333	2.075 ± 0.442	−0.400	0.060
	II	10	2.725 ± 0.629	2.400 ± 0.459		
Psychosomatic problems	I	10	1.475 ± 0.546	1.225 ± 0.362	−0.001	0.990
	II	10	1.400 ± 0.394	1.300 ± 0.405		
Impulsive–hyperactivity behaviors	I	10	2.450 ± 0.575	2.025 ± 0.381	−0.162	0.500
	II	10	2.575 ± 0.708	2.225 ± 0.583		
Anxiety	I	10	2.050 ± 0.497	1.700 ± 0.422	−0.325	0.250
	II	10	2.350 ±0.851	2.050 ± 0.725		

N = the amount of data; SD = standard deviation; *p* = significance value. * *p* < 0.05 (significant).

## Data Availability

The datasets used and/or analyzed during the present study are available from the corresponding author on reasonable request.
